# Positron Emission Tomography (PET) Quantification of GABA_A_ Receptors in the Brain of Fragile X Patients

**DOI:** 10.1371/journal.pone.0131486

**Published:** 2015-07-29

**Authors:** Charlotte D’Hulst, Inge Heulens, Nathalie Van der Aa, Karolien Goffin, Michel Koole, Kathleen Porke, Marc Van De Velde, Liesbeth Rooms, Wim Van Paesschen, Hilde Van Esch, Koen Van Laere, R. Frank Kooy

**Affiliations:** 1 Department of Medical Genetics, Antwerp University, Antwerp, Belgium; 2 Department of Medical Genetics, University Hospital Antwerp, Antwerp, Belgium; 3 Division of Nuclear Medicine and Molecular Imaging, University Hospitals Leuven, Leuven, Belgium; 4 Department of Neuropsychology, University Hospitals Leuven, Leuven, Belgium; 5 Department of Anaesthesiology, University Hospitals Leuven, Leuven, Belgium; 6 Department of Neurology, University Hospitals Leuven, Leuven, Belgium; 7 Center for Human Genetics, University Hospitals, Leuven and KULeuven, Leuven, Belgium; CNRS UMR7275, FRANCE

## Abstract

Over the last several years, evidence has accumulated that the GABA_A_ receptor is compromised in animal models for fragile X syndrome (FXS), a common hereditary form of intellectual disability. In mouse and fly models, agonists of the GABA_A_ receptor were able to rescue specific consequences of the fragile X mutation. Here, we imaged and quantified GABA_A_ receptors *in vivo* in brain of fragile X patients using Positron Emission Topography (PET) and [^11^C]flumazenil, a known high-affinity and specific ligand for the benzodiazepine site of GABA_A_ receptors. We measured regional GABA_A_ receptor availability in 10 fragile X patients and 10 control subjects. We found a significant reduction of on average 10% in GABA_A_ receptor binding potential throughout the brain in fragile X patients. In the thalamus, the brain region showing the largest difference, the GABA_A_ receptor availability was even reduced with 17%. This is one of the first reports of a PET study of human fragile X brain and directly demonstrates that the GABA_A_ receptor availability is reduced in fragile X patients. The study reinforces previous hypotheses that the GABA_A_ receptor is a potential target for rational pharmacological treatment of fragile X syndrome.

## Introduction

Fragile X syndrome patients show mild to severe cognitive impairment and have a distinct physical appearance [1]. Many behavioral problems, such as hyperactivity, aggression, social anxiety, impaired sensorimotory gating and autistic-like features, are associated with the syndrome [2]. In addition, an increased risk of epileptic seizures is reported [3]. Fragile X syndrome is caused by mutations in the *FMR1*-gene, which is characterized by an expansion of a CGG triplet in the 5’ UTR. Normal individuals have 6–44 CGG repeats, while carriers of gray zone or premutation alleles have 45–54 and 55–200 repeats, respectively. Gray zone and premutation alleles inherit unstable and the latter category has a strong tendency to expand to the full mutation range (>200 repeats) upon maternal transmission. *FMR1* silencing is the consequence of rather complex epigenetic modifications. The mRNA containing the elongated CGG repeat has been demonstrated to bind to its own promotor during cellular differentiation, resulting in transcriptional silencing of the gene and loss of the protein FMRP in carriers of the methylated full mutation [4]. FMRP is an RNA-binding protein involved in RNA transport from the nucleus to the dendrites where it is known to regulate translation of proteins important for synaptic development and plasticity (reviewed in [5, 6]).

Previously, we and others have shown a decreased mRNA expression of specific GABA_A_ receptor subunits and other components of the GABAergic system in brain tissue from mouse and fly models for fragile X syndrome (reviewed in [7]). GABA_A_ receptors are the major inhibitory receptors in the mammalian brain implicated in anxiety, depression, epilepsy, insomnia, and learning and memory [8], processes also disturbed in FXS. Ionotropic GABA_A_ receptors are heteropentameric assemblies of 5 subunits, which are chosen out of 19 identified subunits in mammals: α_1–6_, β_1–3_, γ_1–3_, δ, ε, ρ_1–3_, θ and π [9] and which are unlinked to the FXS causative *FMR1* gene. Treatment with agonists of the GABA_A_ receptor has been shown effective in animal models [10–14]. Before treatment on patients can be initiated, it is important to know whether the abnormalities observed in animal models are also detectable and quantifiable in patients. Studies on isolated tissues are not feasible on fragile X patients due to a scarcity of post-mortem materials. To determine whether the GABAergic system is affected in fragile X patients, we quantified the GABA_A_ receptor distribution *in vivo* using positron emission tomography (PET). PET is a functional imaging technology that provides a non-invasive *in vivo* assessment and quantification of receptor binding through administration of selective radio ligands that target specific receptors [15, 16]. In this study, we used [^11^C]flumazenil, a known high-affinity and specific radio ligand of the GABA_A_ receptor, to visualize the receptor occupancy in human FXS brain. Parametric binding potential images of 10 fully characterized male fragile X patients were compared with those of 10 healthy volunteers.

## Materials and Methods

### Human volunteers and study design

This study was approved by the medical ethics committee of the University of Antwerp and the KULeuven. Written informed consent was obtained from each fragile X patient, or his legal guardian, and from each healthy control before research participation. The study was conducted in accordance with statutes and regulations regarding the protection of the right and welfare of human subjects’ participation in biomedical research (World Declaration of Helsinki). Ten healthy male subjects (mean age 35.9, range: 24–51 years) and 10 male fragile X patients (mean age 38.1, range: 23–53 years) were enrolled (S1 Table). The absence/presence of the fragile X mutation was confirmed in all participants, using a CGG-repeat PCR and Southern Blotting on DNA isolated from blood. All subjects fulfilled strict inclusion and exclusion criteria. Inclusion criteria were: male subjects, in good health (as confirmed by physical examination and routine laboratory analysis), and within the age range of 16–55 years of age. Exclusion criteria were: drug/medication abuse, hypersensitivity to medication or flumazenil or benzodiazepines or lidocain, any history of neurological disorders or any other major internal disorder, history of psychiatric disorders. Consumption of any medication or over the counter drug between 2 weeks before or after the study was not allowed. In this regard, only one patient (patient 6) took medication, a spasmolyticum (1 Kemadrin 5mg/day), a benzodiazepine (3/4 Rivotril 2mg/day) and antipsychotica (3 Dipiperon 40mg/day and 1 Largactil 50mg/day) at the time of recruitment. However, medication intake was stopped without major behavioral implications under supervision of a medical doctor. Current heavy smokers (defined as more than 5 cigarettes/day), or heavy smokers who quit less than 3 months before the study, were excluded and consumption of no more than 3 cups caffeine-containing beverages/day (coffee, tea, coke) was allowed. Alcohol usage was prohibited between 2 days before and after study. For radioprotection purposes, previous participants in studies using ionizing radiation during previous 12 months were excluded. Controls were recruited along the same criteria. The characteristics of the subject population, including the results of the psychological testing are provided in S1 Table.

### DNA isolation and fragile X screening

DNA isolation from blood was performed using an automated liquid handling system *Multiprobe II plus ex* (Perkin Elmer, Waltham, MA, USA) and the *chemagic magnetic separation module* (Chemagen, Baesweiler, Germany) based on the magnetic beads principle. The length of the CGG-repeat in the 5’ UTR of the *FMR1* gene was measured using a touchdown PCR with the following primers: FRAXA-F: CGGAGGCGCCGCTGCCAGG and FRAXA-R: TGCGGGCGCTCGAGGCCCAG. PCR products were analysed using capillary gel electrophoresis (ABI3130XL, ABI, Foster city, CA, USA). In case of a male fragile X full-mutation the CGG-expansion is >200 repeats and the PCR will not be able to amplify the repeat. Consequently, large CGG-repeats and methylation status at the level of the *FMR1* gene were investigated using Southern Blot with *HindIII/EagI* (New England Biolabs, Ipswich, MA, USA) as described [17].

### Neurological assessment

All fragile X patients were subjected to a standard neurological examination, including higher mental functions, cranial nerves, meningeal stimulation signs, locomotion, sensibility, reflexes, coordination and motion by a board certified neurologist. Fragile X patients as well as healthy controls were subjected to an EEG.

### Neuropsychological assessment

All healthy controls and fragile X patients were subjected to a neuropsychological examination, the Wechsler Adult Intelligence Scale (WAIS III), by a certified psychologist (S1 Table). Correlations with GABA_A_ receptor binding potential values (volume of interest-based comparisons) were made with the scaled values for full scale IQ (TIQ), verbal IQ (VIQ) and performance IQ (PIQ) and with the scaled values of the four index scores (verbal comprehension (VCI), perceptual organization (POI), working memory (WMI) and processing speed (PSI)). To avoid floor effects, the scaled IQ scores are age-corrected, showing a correct measure of the capability of the patients.

### Radio ligand preparation

PET imaging was performed using the tracer [^11^C]flumazenil, a ligand that binds to α subunits of the GABA_A_ receptor. The routine synthesis of [^11^C]flumazenil was performed as described previously [18].

### Anaesthesia

Fragile X patients were anesthetized before PET scanning using dexmedetomidine (Precedex, Hospira, Inc., USA). Sedation was initiated with a bolus dose of 0.5–1 μg/kg and maintained by a continuous infusion of dexmedetomidine (0.25 μg/kg/h). Two healthy controls underwent scanning with and without dexmedetomidine to evaluate *in vivo* differences on the GABA_A_ binding potential of flumazenil.

### PET imaging

For each subject, a bolus of on average 265.4 MBq (SD 45.8) of [^11^C]flumazenil was injected through an intravenous catheter. Simultaneously, a dynamic emission scan was started in three-dimensional mode, consisting of 21 frames with progressive frame duration (4x15s, 2x30s, 3x1min, 2x2.5 min, 10x5min). Total scan duration was 60 min. PET acquisitions were performed using a HiRez Biograph 16 PET-CT camera (Siemens, Erlangen, Germany). Images were reconstructed with a standard three-dimensional filtered-back protection (3DRP) algorithm, including scattered and measured attenuation correction. The resulting transverse and axial spatial resolution is approximately 5mm. Dynamic data were motion corrected and spatially normalized to MNI (Montreal Neurological Institute) space (PMOD 2.95, Zurich, Switzerland). A voxel-based reference tissue model approach was used (Ichise model, MRTM2, PMOD v2.9, PMOD Inc, Zurich) to calculate binding potential (BP_ND_) [19] parametric maps with the pons as reference region [20]. On the spatially normalized maps, a predefined VOI analysis was performed. The same VOI definition, based on Brodmann areas, was used as in previous studies, where larger cortical VOIs were defined in order to reduce type I errors [21]. The following VOIs were measured: frontal, temporal, mesial temporal, parietal, occipital, central and cingulate cortical brain regions and the thalamus. To account for slight differences in subcortical VOI placement due to spatial normalisation, the subcortical VOIs were manually adjusted to the activity images for those areas by means of shifting and scaling when needed. To exclude that demographic variables and PET injection parameters are the cause of the differences in BP values, correlations between the group BP values and age, weight, injection activity and specific activity of the flumazenil tracer were made. No correlations were found.

### Statistical analysis

Statistical calculations were performed using SPSS. Independent samples t-test was used to compare BP_ND_ values between groups. BP_ND_ values were also expressed in z-scores to indicate the number of standard deviations by which the measured BP for any given subject differed from the mean. Z-scores were calculated by subtracting the mean population BP for the total grey matter (BP_ND_ group_AVG) from the observed total grey matter BP per subject and dividing by the standard deviation of the population BP (BP_ND_ group_SD). Pearson correlations were made to study the relationship between BP_ND_ values and IQ values. Correlative results were not corrected for multiple testing but were reported as exploratory. Uncorrected *p*-values are given for descriptive reasons only.

## Results

All participants, fragile X patients and healthy volunteers, were screened for the fragile X full mutation. All patients showed a fully methylated full mutation in blood, and mosaicism was not detected on Southern blot. All volunteers showed a single band in the unaffected size range for FXS, indicating absence of fragile X syndrome or any of the premutation related disorders. All patients and healthy volunteers underwent standard neurological examination, and no specific clinical neurological abnormalities were detected. All study participants were subjected to electroencephalography (EEG). No gross abnormalities or epileptic discharges were observed.

### PET imaging

**Fig 1:**
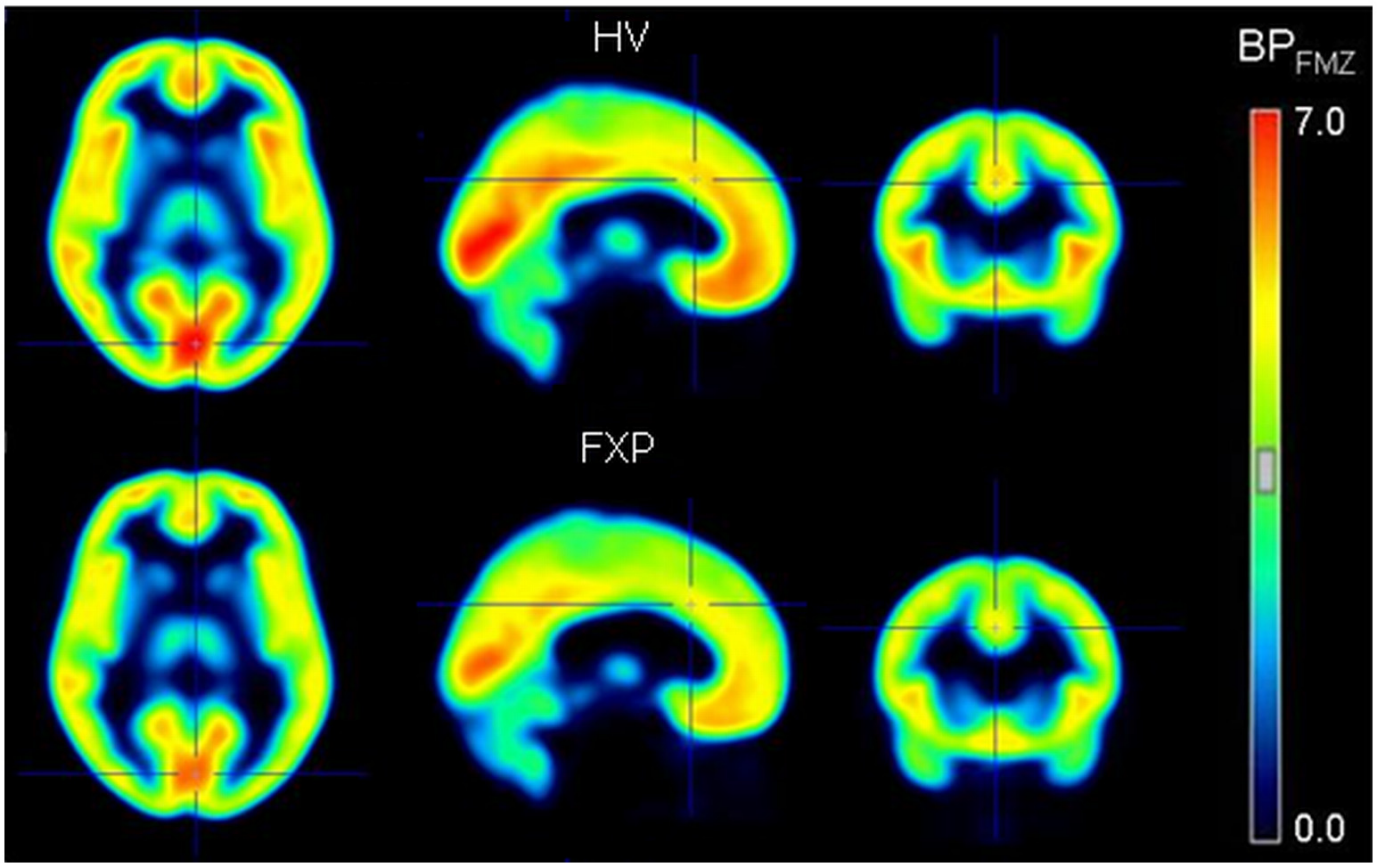
Transversal, sagittal and coronal slices of GABA_A_ receptor PET images expressed in [11C]flumazenil binding potential (BPFMZ) values, averaged for the 10 healthy controls (HV, upper panel) and the 10 fragile X patients (FXP, lower panel). BPND values are indicated by the color bar.

**Fig 2:**
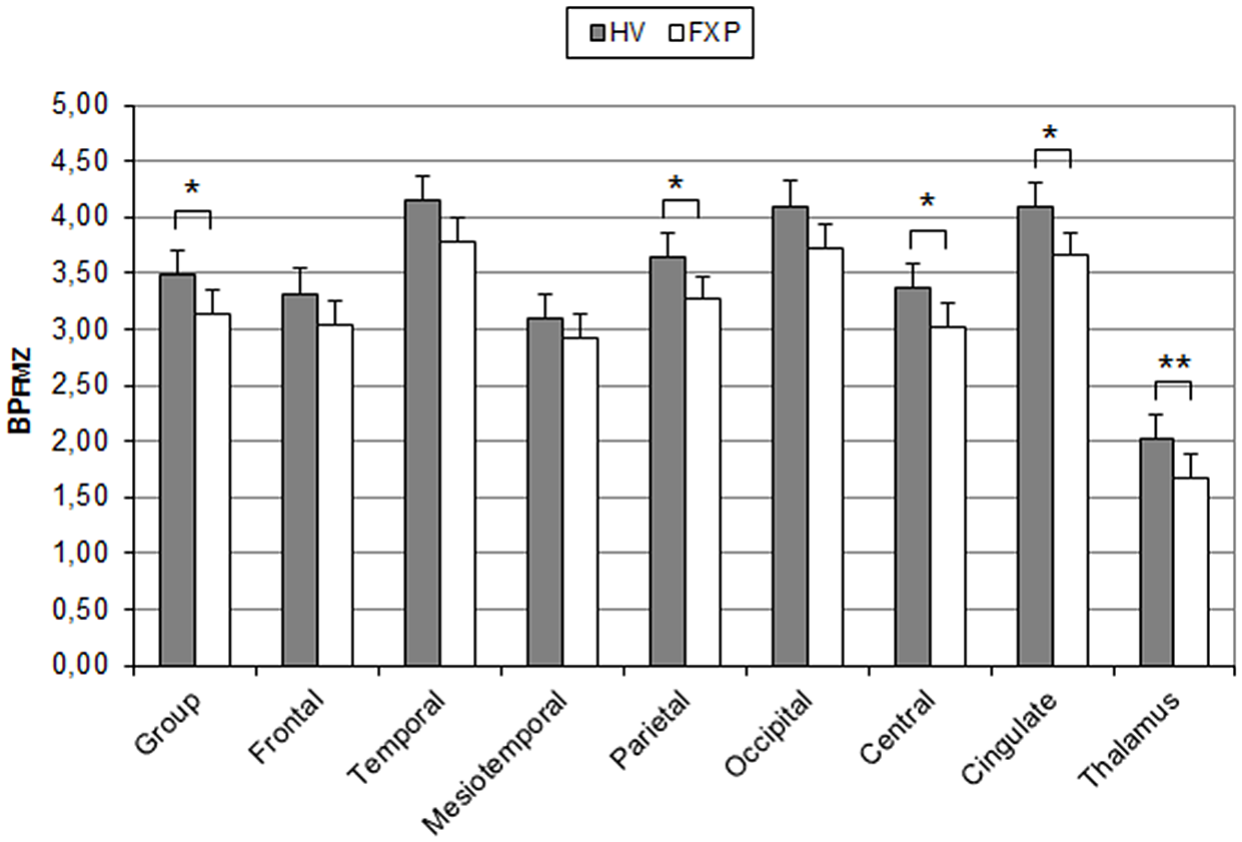
Bar chart of GABA_A_ receptor availability expressed in mean binding potential (BP) values of [11C]flumazenil. GABA_A_ receptor availability is significantly decreased in the fragile X patient group (FXP) compared to the control group (healthy volunteers, HV) for the regions indicated with * (*: p<0.05, **: p<0.01). Group represents the composite VOI resulting from the weighted sum of all VOIs in the study, and includes total grey matter. Error bars indicate SEM (n = 10/group). VOIs represented include all cortical regions and the thalamus. Cerebellum and striatum are not included.

[^11^C]flumazenil PET scans were performed on 10 fully characterized male FXS patients (Age 35.9 ± 3.7) and 10 healthy controls (Age 38 ± 3.7, for inclusion- exclusion criteria, see Materials and Methods section). Given the impact of the experimental procedure and the high incidence of anxiety amongst FXS subjects, patients were lightly sedated with dexmedetomidine. Dexmedetomidine has sedative, analgesic, sympatholytic, and anxiolytic properties and is relatively unique in its ability to provide sedation without causing respiratory depression. It was selected as, in contrast to most anaesthetics exerting their action through GABA_A_ receptors, the mechanism of action of dexmedetomidine is agonism of α_2_-adrenergic receptors. By scanning two healthy volunteers twice, once with and once without dexmedetomidine sedation we confirmed this anaesthetic does not interfere with the outcome of our study. The non-displaceable binding potential (BP_ND_; the free plus non-specifically bound concentration in brain), which reflects the availability of GABA_A_ receptors, showed a significant mean decrease of 10% (BP_ND_ group_AVG = 3.31, BP_ND_ HV = 3.48, BP_ND_ FXP = 3.14; p = 0.036; with z-scores ranging from -0.36 to 1.41 for the controls and -1.83 to 1.20 for the patients) in total grey matter binding of flumazenil in FXS patients when compared to the controls (Figs 1 and 2; S1 Fig). The decrease ranged from 6–17% in different regions of the brain but was fairly homogeneous. Regionally, the decrease in BP_ND_ was significant in the parietal (BP_ND_ parietal_AVG = 3.45, BP_ND_ HV = 3.64, BP_ND_ FXP = 3.26; *p* = 0.038), central (BP_ND_ central_AVG = 3.19, BP_ND_ HV = 3.36, BP_ND_ FXP = 3.02; *p* = 0.048) and cingulate (BP_ND_ cingulate_AVG = 3.87, BP_ND_ HV = 4.09, BP_ND_ FXP = 3.65; *p* = 0.016) cortical regions, and in the thalamus (BP_ND_ thalamus_AVG = 1.84, BP_ND_ HV = 2.02, BP_ND_ FXP = 1.67; *p* = 0.001).

Five patients showed BP_ND_ values within the range of the controls and 5 showed ranges below these values (S2 Fig). It is striking that the intergroup variance in BP_ND_ values within the patient group is significantly larger than the variance within the control group (Levene’s test, *p*<0.05, data not shown). This observation indicates that factors other than the fragile X mutation may potentially influence flumazenil binding to the receptor, as previously suggested by FXS modifier genes [22]. Alternatively, the high intergroup variance may indicate mosaicism for the presence of a full mutation and a permutation in the brain. Although we excluded mosaicism in the blood of our patients, mosaicism in other tissues can never be excluded.

### Cognitive testing

**Fig 3:**
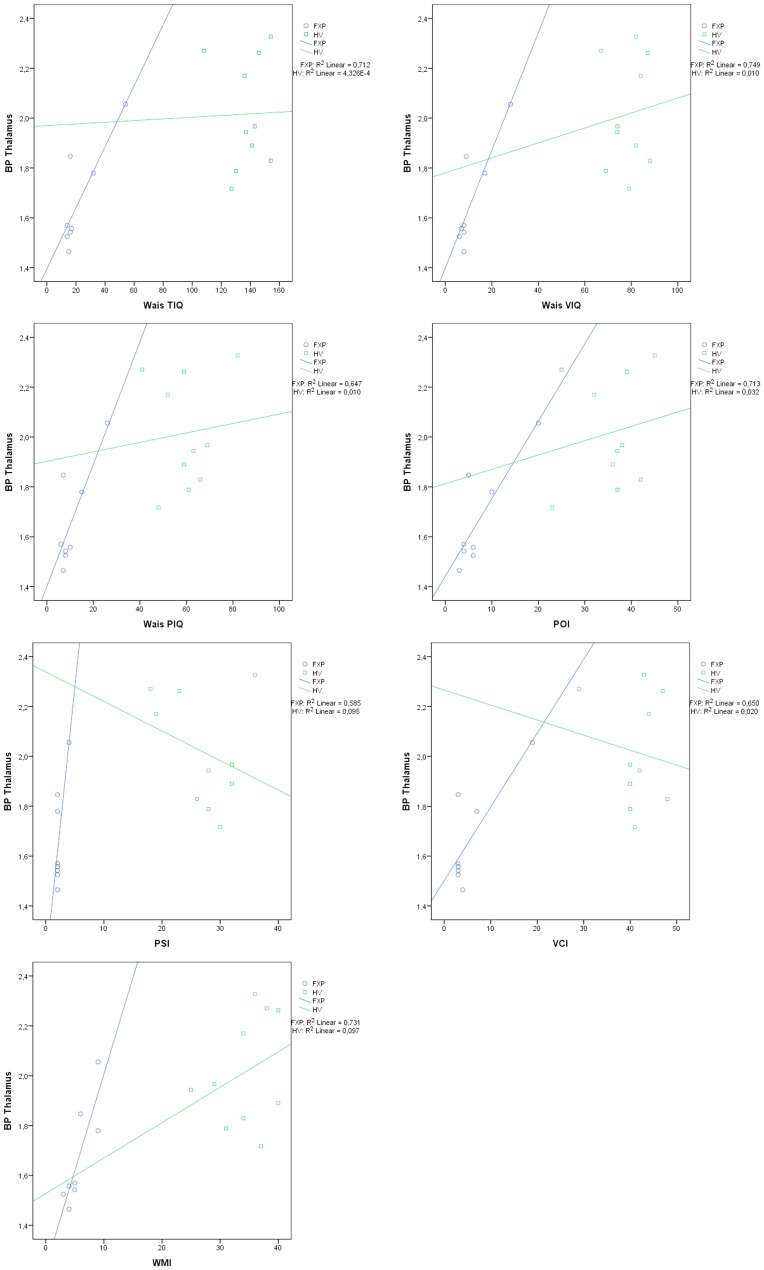
Regression plot of IQ (TIQ: total IQ, VIQ: verbal IQ, PIQ: performance IQ; Scaled IQ values) and SCALED index scores (VCI: verbal comprehension index, POI: perceptual organization index, WMI: working memory index, PSI: processing speed index) against thalamic BPND.

The cognitive ability of patients and healthy controls was examined with the WAIS III test (S1 Table). The full scale IQ score (TIQ) of the FXS patients was 50 ± 2 (mean ± S.E.M.). There was no significant difference between mean verbal IQ (VIQ), which measures the verbal, crystallized abilities, and mean performance IQ (PIQ), which measures the nonverbal, fluid abilities, with a score of 48 ± 2 and 49 ± 3, respectively. The TIQ can be subdivided into four index scores: verbal comprehension (VCI), perceptual organization (POI), working memory (WMI), and processing speed (PSI). These four mean index scores were obtained for 8 patients and were 55 ± 3 (VCI), 55 ± 3 (POI), 52 ± 1 (WMI) and 51 ± 1 (PSI), respectively. These scores agree with earlier observations and confirm that there were no specific weaknesses or strengths in any of the index domains. It is remarkable that the patient showing the highest total BP_ND_ value also has the highest IQ (69). Therefore, we correlated the [^11^C] flumazenil BP_ND_ values with the scaled IQ scores and index scores in patients and control subjects and found a strong correlation between the BP_ND_ of thalamus and all IQ sub scores in patients (TIQ: r = 0.844; VIQ: r = 0.866; PIQ: r = 0.804) and index scores (VCI: r = 0.806,; POI: r = 0.845; WMI: 0.855; PSI: r = 0.765) (Table 1 and Fig 3). No relevant correlations were found between IQ scores and BP_ND_ values in the healthy controls. It is important to note that this pilot study is not sufficient to draw significant statistical conclusions concerning a possible correlation between GABA_A_ receptor availability and IQ scores and further expansion of our patient cohort is necessary.

**Table 1:** Volume of interest-based correlation between FMZ BP and IQ and index scores for the healthy volunteers and fragile X patients. Abbreviations: TIQ: total IQ score, VIQ: verbal IQ, PIQ: performance IQ, VCI: verbal comprehension index, POI: perceptual organization index, WMI: working memory index, PSI: processing speed index.

	Healthy Volunteers (n = 10)							Fragile X Patients (n = 8)						
	TIQ	VIQ	PIQ	VCI	POI	WMI	PSI	TIQ	VIQ	PIQ	VCI	POI	WMI	PSI
Group	-.198	-.124	-.044	-.354	-.022	.187	-.243	.491	.509	.462	.561	.483	.359	.622
Frontal	-.181	-.083	-.061	-.348	-.018	.225	-.262	.467	.481	.443	.545	.459	.313	.618
Temporal	-.317	-.183	-.166	-.450	-.140	.342	-.278	.434	.454	.404	.523	.419	.282	.596
Mesial temporal	-.376	-.092	-.312	-.479	-.325	.413	-.392	.427	.438	.407	.476	.420	.319	.514
Parietal	-.219	-.061	-.120	-.351	-.145	.207	-.188	.474	.487	.451	.523	.481	.367	.569
Occipital	-.391	-.439	-.101	-.619	-.111	-.012	-.253	.486	.486	.477	.567	.508	.278	.648
Central	.005	-.001	.122	-.251	.044	-.019	-.030	.516	.518	.504	.562	.544	.372	.611
Cingulate	-.234	.044	-.234	-.282	-.148	.341	-.442	.531	.558	.491	.601	.516	.420	.658
Thalamus	.021	.098	.098	-.141	.180	.311	-.309	.844	.866	.804	.806	.845	.855	.765

## Discussion

PET may be a key technology in identifying the physiological consequences of gene mutations associated with intellectual disabilities [23]. The utility of PET in elucidating the functional consequences of specific genetic abnormalities was previously reported in the Angelman and Prader-Willi syndromes. Both diseases result from a maternal and paternal deletion, respectively, of an imprinted region on chromosome 15q11-q13, encoding the GABA_A_ receptor subunit genes *β*_*3*_, *α*_*5*_ and *γ*_*3*_ [24, 25]. Six Prader-Willi patients showed a 14% global decrease in [^11^C]flumazenil binding [26], while a significantly decreased GABA_A_ receptor occupancy was observed in specific brain regions of Angelman patients [27]. These observations demonstrate that a deletion of specific subunits may result in a measurable reduction of the amount of GABA_A_ receptors in the brain. Even though we have no information on the expression alterations of individual subunits of the GABA_A_ receptor in FXS patients, the data in these deletion patients suggest that a reduction in expression of of 8 GABA_A_ receptor subunits on the mRNA level, as observed in FXS animal models, can have a significant impact on the amount of receptor protein in the brain.

To assess the functional consequences of a reduced amount of GABA_A_ receptor mRNA and protein observed in fragile X animal models (reviewed in [7]) binding levels of the radioligand [^11^C] flumazenil to the GABA_A_ receptor in the brain of fragile X patients were investigated using PET. We observed a significant decrease of 6-17% in brain GABA_A_ receptor availability in the patient cohort. This decrease may even be an underestimation of the effect in the total fragile X patient population. First of all, an important difference between fragile X patients and controls is that mild sedation with a α_2_-adrenergic agonist, dexmedetomidine, was only used in the patient group. To estimate the potential bias of dexmedetomidine anaesthesia on the [^11^C]flumazenil BP_ND_ in patients *in vivo*, two healthy controls were scanned twice on two consecutive days, the first day without and the second day with dexmedetomidine sedation. We observed a mild increase (4.9–6.0%) in grey matter BP_ND_ after sedation, suggesting a negative confound on the observed effect between patients and controls. Studies in animal models may further answer this question. Secondly, only mildly affected fragile X patients could be enrolled in this study because of the impact of the experimental protocol on the patient. This selection may have created a bias and GABA_A_ receptor occupancy values may be even more decreased, had we been able to measure a more severely affected group of patients. Lastly, flumazenil is an antagonist that binds to the benzodiazepine binding site of the GABA_A_ receptor and as a consequence only benzodiazepine sensitive GABA_A_ receptors are measured. For instance, occupancy of the δ-containing extrasynaptic GABA_A_ receptors is not recorded. This is the most significantly under expressed receptor subunit in fragile X mice on the RNA level [28, 29]. Measurement of all receptor subtypes might reveal even larger differences between patients and controls. An alternative explanation for the decreased BP_ND_ values is that the receptor affinity for flumazenil, rather than the density, is altered in FXS patients. Flumazenil interacts with the benzodiazepine binding site, which is composed of an α_1_,_2_,_3 or 5_ subunit together with a β_n_ and γ_2_ subunit. We previously found decreased expression of the *α*_*1*_,_*3*_, *β*_*1*_,_*2*_ and *γ*_*2*_ mRNA without compensatory up regulation of the remaining subunits in the FXS mouse, suggesting that the amount of benzodiazepine binding site containing subtypes of the receptor are reduced in the mouse model [28].

All patients were neuropsychologically tested and it is remarkable that the patient showing the highest total BP value in the range of the controls also has the highest IQ, a score of 69 (S2 Fig). When we correlated the flumazenil BP with the scaled IQ and index scores, we found that the BP value of the thalamus was positively correlated with the IQ scores and index scores in the patient but not in the control group. The thalamus has not been previously implicated in the fragile X syndrome pathology, apart from neuroanatomical studies that demonstrated an increased volume of this brain structure in women and children (20–22). However, it's important to note that the size of our cohort in this pilot study is not sufficient to draw significant statistical conclusions concerning a possible correlation between GABA_A_ receptor availability and IQ scores and studies on a much larger cohort of patients are necessary to confirm such a possible correlation. In this regard, it is important to mention that the sample size required reaching 80% power with a significance level lower than 0.01 is 56 patients. Given the difficulty of recruiting patients for this study, which includes a full day of neurological and neuropsychological testing and a PET scan, we concluded that a study of this size is logistically not feasible with fragile X patients, and decided to perform this study with a smaller sample size of n = 10.

GABA_A_ receptors are the major inhibitory neurotransmitter receptors in the mammalian brain implicated in anxiety, depression, epilepsy, insomnia, and learning and memory [8], processes disturbed in fragile X syndrome. Diminished expression of the GABA_A_ receptor in fragile X syndrome may thus have functional consequences that relate to the behavioral and epileptic phenotype associated with fragile X syndrome [30]. Using PET, we were able to validate *in vivo* a reduction in the amount of the GABA_A_ receptor as previously observed in mice and fly models. This is the first time that a potential target for treatment of fragile X syndrome has been visualized directly in the brain of patients.

Currently, FXS patients are prescribed various drugs that are aimed at symptomatic treatment and targeted treatment is lacking. Most recently, increased insights into the pathophysiology of FXS have raised hopes for the patient [30–33]. Two receptor systems emerged as the most promising targets for drug treatment, including the mGluR5 receptor and the GABA_A_ receptor. Enhanced signalling through mGluR5 is hypothesized to be responsible for cognitive and behavioral deficits in fragile X patients [34]. Trials dampening mGluR signalling in human patients, either by acting directly on the receptor or indirectly by stimulating the metabotropic GABA_B_ receptor, which results in the repression of glutamate release from presynaptic terminals, were reported at least partially effective in the most severely affected patient group in one study were the patients were treated with AFQ056 [35] and in the group with the highest social withdrawal in a second study were the patients were treated with STX 209 [36]. Deficits in GABA_A_ receptor signalling are equally apparent in the disorder [30]. Pharmacological approaches targeting the GABA_A_ receptor in the FXS mouse model have been successful in correcting amygdala-based symptoms [14], in preventing epileptic seizures [11] and in correcting multiple behavioural abnormalities [12–14]. Patient trials using ganaxolone, a neurosteroid drug targeting the GABA_A_ receptor have been initiated (www.clinicaltrials.govNCT01725152). In addition to visualizing for the first time GABA_A_ receptor occupancy in brain of FXS patients, our study also predicts that some patients might respond better than others to treatment with drugs targeting the GABA_A_ receptor, a phenomenon also observed in previous clinical trials targeting the glutamatergic system [35, 36]. Our results indicate that in some patients the target receptors are more compromised than in others. As such, the GABA_A_ receptor occupancy quantification using [^11^C]flumazenil may have the potential to serve as a biomarker to predict the efficacy of treatment with drugs targeting this receptor in FXS.

## Supporting Information

S1 Fig BP_ND_ values for the total grey matter expressed in z-scores for healthy controls (HV) and fragile X patients (FXP). (TIF)

S2 Fig The cognitive ability of patients and healthy controls as determined using WAIS-III (TIQ), and their corresponding BP_ND_ group values. (DOC)

S1 Table Subject population and psychological testing. (DOC)
