# Supplementary figures and images for: Positron Emission Tomography (PET) Quantification of GABAA Receptors in the Brain of Fragile X Patients
Source: PLoS One. 2015 Jul 29;10(7):e0131486. doi: 10.1371/journal.pone.0131486 (PMC4519313; doi:10.1371/journal.pone.0131486)

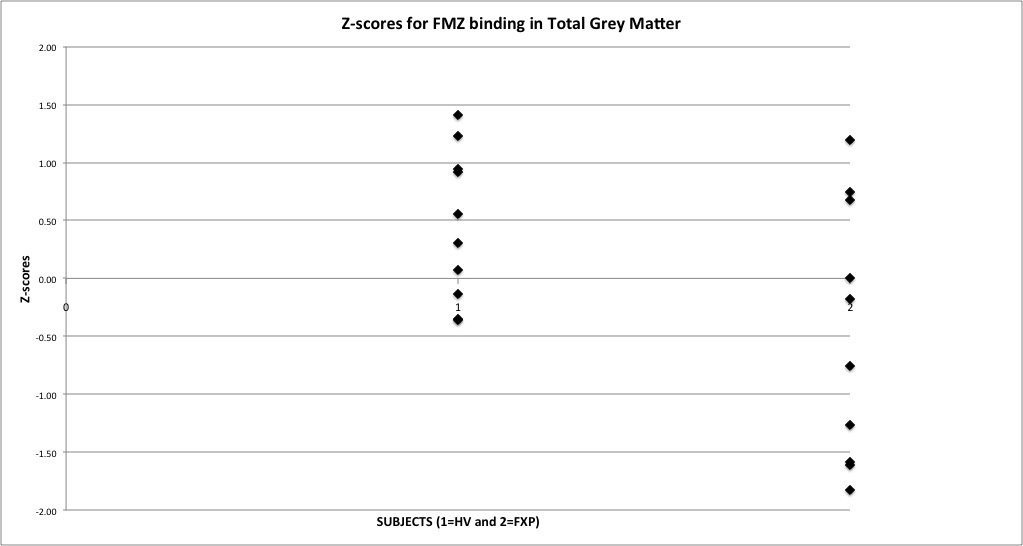

Supplement: S1 Fig — (TIF) [file pone.0131486.s001.tif]
