# Supplementary material for: Positron Emission Tomography (PET) Quantification of GABAA Receptors in the Brain of Fragile X Patients
Source: PLoS One. 2015 Jul 29;10(7):e0131486. doi: 10.1371/journal.pone.0131486 (PMC4519313; doi:10.1371/journal.pone.0131486)
Supplement: S2 Fig — (DOC) [file pone.0131486.s002.doc]

**Supplementary Table 1.** Subject population and psychological testing

|  | Healthy Volunteers  (n=10) | Fragile X Patients (n=10) |
| --- | --- | --- |
| Age (y) | 35.9 ± 3.7 | 38.1 ± 3.7 |
| Wais TIQ | 117 ± 3 | 50 ± 2 |
| Wais VIQ | 118 ± 3 | 48 ± 2 |
| Wais PIQ | 112 ± 4 | 49 ± 3 |
| VCI | 121 ± 3 | 55 ± 3 |
| POI | 110 ± 5 | 55 ±3 |
| WMI | 108 ± 3 | 52 ± 1 |
| PSI | 121 ± 5 | 51 ± 1 |

Values represent means ± SEM. Abbreviations: TIQ: total IQ score, VIQ: verbal IQ, PIQ: performance IQ, VCI: verbal comprehension index, POI: perceptual organization index, WMI: working memory index, PSI: processing speed index
